# Lesion shrinkage and tooth preservation-based optimal timing for definitive surgery following marsupialization of cystic odontogenic jaw lesions

**DOI:** 10.4317/medoral.27585

**Published:** 2026-01-24

**Authors:** Jeong-Rae Seo, Eunji Park, Yeong-Cheol Cho, Iel-Yong Sung, Jang-Ho Son

**Affiliations:** 1 Department of Oral and Maxillofacial Surgery, University of Ulsan Hospital, University of Ulsan College of Medicine, Ulsan, Republic of Korea; 2 Department of Dentistry, University of Ulsan Hospital, University of Ulsan College of Medicine, Ulsan, Republic of Korea; 3 Department of BigData Center of Ulsan University Hospital, University of Ulsan College of Medicine, Ulsan, Republic of Korea; 4 Department of Dentistry, University of Ulsan Hospital, University of Ulsan College of Medicine, Ulsan, Republic of Korea; 5 Department of BigData Center of Ulsan University Hospital, University of Ulsan College of Medicine, Ulsan, Republic of Korea

## Abstract

**Background:**

This study aimed to investigate the appropriate timing for the second enucleation surgery to remove entire cystic odontogenic jaw lesions based on tooth preservation and lesion shrinkage patterns after the marsupialization of jaw lesions.

**Material and Methods:**

This retrospective study included 20 patients who underwent marsupialization between January 2013 and December 2024. Changes in the number of lesions affecting the teeth before and after marsupialization were investigated, and two-dimensional lesion size changes on panoramic radiographs and three-dimensional volume changes on computed tomography images over time were measured and analyzed. Statistical analysis was performed using the Mann-Whitney test for non-normally distributed, continuous variables and the chi-square test or Fisher's exact test for nominal variables, as appropriate, with a significance level of p&lt;0.05.

**Results:**

After an average of 62.51±31.17 weeks of marsupialization, the number of lesion-involved teeth decreased by 46.48%. An average lesion reduction of 50.85±14.95% and 62.86±17.25% (p&lt;0.05) in two- and three-dimensional volumetric assessments was observed. Lesion shrinkage was the most significant between 16 and 24 weeks (95% confidence interval), with a mean of 18±10 weeks.

**Conclusions:**

Definitive enucleation surgery should be delayed by 24 weeks to maximize neo-bone formation in the cystic odontogenic jaw lesion cavity and maintain the vitality of the affected teeth.

## Introduction

The management of cysts or cystic lesions of the jaw has been widely debated (1 , 2). For jaw cystic lesions, conservative surgical management includes marsupialization, advocated by Partsch in 1892, and decompression, proposed by Thoma in 1958 (3). These methods can reduce damage to adjacent anatomical structures and preserve bone and involved teeth through shrinkage and substantial reduction in lesion size (4). Substantial evidence supports marsupialization/decompression as a versatile treatment option with low complication rates for managing odontogenic/non-odontogenic cysts and unicystic odontogenic jaw tumors (5 - 7). Although some studies have used these conservative techniques as sole treatments (8), a second surgery was necessary in most cases to completely remove pathological tissues (9).

Because conservative approaches depend on releasing intraluminal pressure, which promotes gradual new bone formation over time, patient compliance with the unpredictable and prolonged duration of lesion reduction is an important factor for successful outcomes (10 , 11). Therefore, surgeons can provide further scientific information regarding the benefits to patients over time and the optimal timing for a second surgery.

The current study aimed to investigate the outcome of marsupialization of cystic lesions in the jaw. We hypothesized that marsupialization would preserve the lesion-involved teeth with a lower complication rate and that the appropriate timing for a second enucleation surgery could be predicted to remove the entire lesion. To address the hypotheses, we investigated the reduction rate of the number of lesion-involved teeth and the pattern of lesion shrinkage in relation to the patient's age, histological types, initial size, and sites of the lesion, as well as the number of involved teeth over time.

## Material and Methods

Study design and sample size

To address the research objectives, a retrospective study was designed and implemented. The study population included 20 patients who underwent marsupialization due to large cystic jaw lesions with a potential risk of inferior alveolar nerve injury, oronasal or oroantral fistula formation, or multiple tooth extractions or loss of pulp vitality at the Department of Oral and Maxillofacial Surgery, Ulsan University Hospital, between January 2013 and December 2024. The inclusion criteria for the study were as follows: Availability of medical records, panoramic radiographs, and multidimensional or cone-beam computed tomography (CBCT) images, performance of an incisional biopsy of the cystic lining during marsupialization to establish a histopathological diagnosis and rule out malignancy, secondary enucleation with peripheral ostectomy of the remaining lesion after marsupialization, follow-ups at approximately 1-month intervals to check whether proper oral hygiene and stromal opening were sustained during the marsupialization period. The study protocol was approved by the Institutional Review Board of Ulsan University Hospital (UUH IRB 2025-03-027). The study was conducted and reported in accordance with the Declaration of Helsinki and STROBE guidelines (12). The requirement for informed consent was waived due to the retrospective nature of the study.

Study variables

The outcome variables included 1) the reduction in the number of lesion-involved teeth and complications, and 2) pattern of lesion shrinkage over time. The number of teeth involved over time was determined using panoramic and computed tomography images. The recurrence rate was evaluated at the final visit after at least 1 year of follow-up. Two-dimensional lesion size changes over time were observed on serial panoramic radiographs using the Sketch-Up software (Trimble Inc., Sunnyvale, CA, USA), and their three-dimensional volume changes over time were measured on multidimensional computed tomography or CBCT images using the OnDemand3D software (Cybermed, Seoul, South Korea). The same observer investigated lesion size and volume on the radiographs thrice, and the mean value was used for analysis. The reliability of repeated measurements was assessed by calculating the intraclass correlation coefficient (ICC). The ICC values indicated excellent reliability (ICC= 0.990 and 0.971 for two- and three-dimensional measurements, respectively; 95% CI: 0.980-0.996 and 0.926-0.991, respectively). The interval speed of shrinkage, defined as the lesion cavity change between every visiting interval, and the trend of lesion change were calculated and represented in graphs. To assess the predictors influencing bone dimensional changes, the patient's age, pathology of the lesion, site of the lesion, initial two-dimensional size and three-dimensional volume of the lesion, and number of teeth involved were analyzed.

Statistical analysis

Treatment data were evaluated using descriptive statistics, including the mean ± standard deviation and frequency. Panoramic and computed tomography images were compared using the Mann-Whitney test for non-normally distributed continuous variables and the chi-square test or Fisher's exact test for nominal variables, as appropriate. Lesion changes at 24 weeks were analyzed using the Wilcoxon signed-rank test. The proportion of patients exhibiting peak lesion shrinkage between 16 and 24 weeks was assessed using the exact binomial test. Additionally, longitudinal changes in the lesion volume over time were analyzed using a generalized linear mixed model to account for repeated measures. Data manipulation and statistical analyses were performed using IBM SPSS Statistics for Windows version 28 (IBM Corp., Armonk, NY) and the R software version 4.2.2 (R Foundation for Statistical Computing, Vienna, Austria; www.r-project.org). The significance level was set at p&lt;0.05.

## Results

This study included a total of 20 patients (15 men and five women), with ages ranging from 31 to 77 years (mean, 47.40±11.74). Histological diagnoses included radicular cysts (n=8), dentigerous cysts (n=8), odontogenic keratocysts (OKC, n=2), and unicystic ameloblastoma (n=2). The mean duration of marsupialization was 62.51±31.17 weeks, after which all patients underwent enucleation as the definitive surgical procedure (Table 1).

Table 1

**Figure 1:**
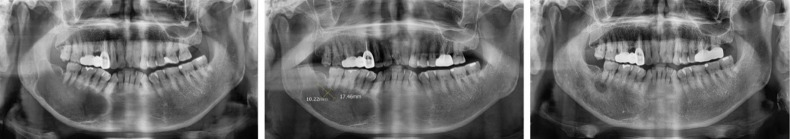
Series of radiographs of lesion-involved teeth before and after marsupialization.Left: Cystic lesion with affected teeth from the mandibular right canine to the second molar and inferior alveolar nerve Middle: Three months after marsupialization, a 17×10-mm window was created. Right: Immediately before definitive enucleation after 118 weeks, the affected neurovascular component and teeth were outside the lesion, except the second molar.

Tooth preservation rates were assessed based on pulp vitality and extraction status before and after marsupialization. Approximately 12, 24, and 36 weeks after marsupialization, the number of teeth within the lesions decreased by 13.44, 26.69, and 30.93%, respectively, and ultimately by 46.48% throughout the mean total treatment period. No invasion or injury to adjacent anatomical structures, including neurovascular components, was observed after definitive surgery (Figure 1).

1

One case of recurrence was identified in a patient with OKC and nevoid basal cell carcinoma syndrome, occurring 8 years after the final surgical removal of the lesion.

**Figure 2:**
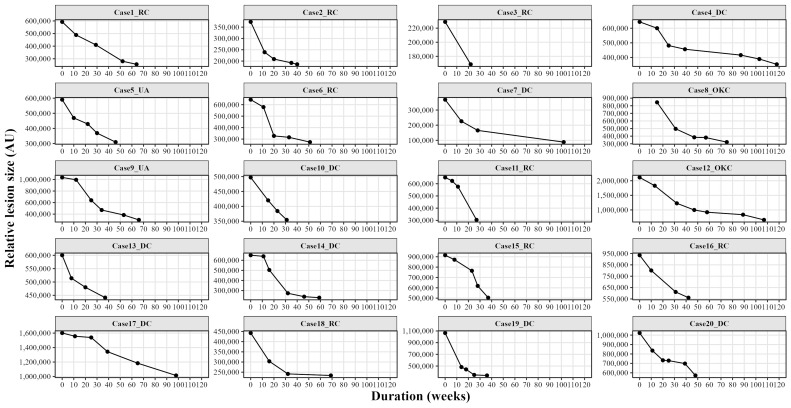
Lesion size changes during marsupialization on panoramic radiographs.

**Figure 3:**
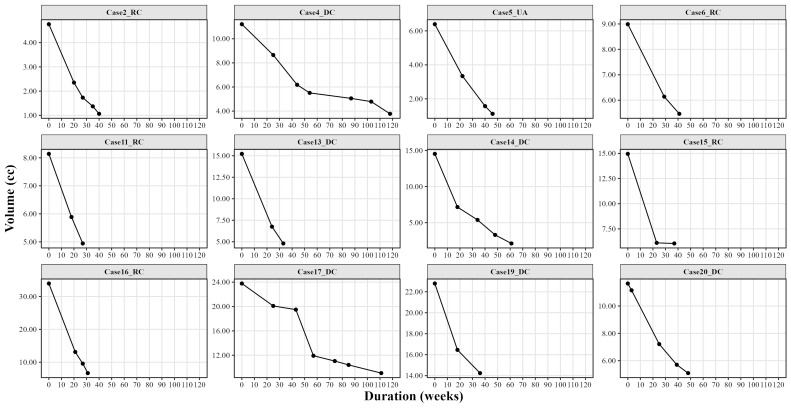
Lesion volume changes during marsupialization on computed tomography images.

Lesion shrinkage was evaluated using both two-dimensional and three-dimensional analyses. Throughout the entire treatment duration, lesions showed an average reduction of 50.85±14.95% (p&lt;0.05) in two-dimensional measurements and an average reduction of 62.86±17.25% (p&lt;0.05) in three-dimensional volumetric assessments, with no statistically significant differences between panoramic radiographs and computed tomography images (Figure 2 and Figure 3).

2

3

**Figure 4:**
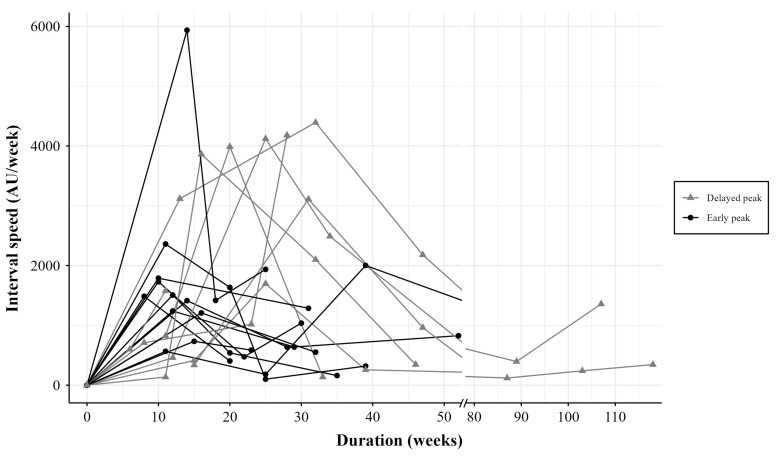
Interval speed patterns of lesion change. Lesion shrinkage was the most significant between 16 and 24 weeks (95% confidence interval), with a mean of 18±10 weeks.

Changes in lesion size and volume were monitored at each follow-up visit to analyze shrinkage patterns and identify the periods of the most pronounced shrinkage. Based on the interval speed of lesion change, lesion shrinkage was the most significant between 16 and 24 weeks (95% confidence interval; mean, 18±10) after marsupialization. Moreover, two distinct patterns were identified: An early peak pattern (n=11), characterized by rapid initial shrinkage (mean, 14.64±8.43 weeks) followed by a gradual decrease with time, and a delayed peak pattern (n=8), characterized by an initially minimal response followed by significant shrinkage at a later time point (mean, 23.50±7.35 weeks) and subsequent deceleration (Figure 4).

4

However, no significant correlations were found between the two shrinkage patterns and patient age (p=0.808), initial two-dimensional lesion size (p=0.342), histological type (p=0.398), presence or absence of cortical bone perforation (p=1.000), or initial number of teeth involved (p=0.511).

## Discussion

Marsupialization is considered a conservative treatment option for large cystic jaw lesions that avoids aggressive radical surgery and adjacent anatomical structures after lesion shrinkage (13 , 14). Some studies with larger sample sizes indicated that marsupialization or decompression is effective in treating medium- or large-sized cysts (15 , 16). In their meta-analysis, Al-Moraissi et al. compared recurrence rates across treatment modalities for OKC and found that marsupialization followed by secondary enucleation with peripheral ostectomy was associated with a lower recurrence rate than either enucleation or marsupialization alone (16). In this study, although adjacent anatomical structures were not invaded or injured, including neurovascular components or oroantral communication, recurrence occurred in one patient with syndromic OKC 8 years after the final surgical removal of the lesion. Considering the high recurrence rate of syndromic OKC (17), long-term follow-up appears to be essential, even after marsupialization. The optimal timing for the second complete removal surgery and the benefits of long-lasting marsupialization periods are still subject to debate (6 , 14 , 18). To ascertain the benefits of marsupialization during periods of lesion shrinkage, we investigated the extent to which involved dental roots could be excluded from the lesion according to marsupialization periods. This is important because, after eliminating the risk of damage to adjacent anatomical structures, preserving the vitality of teeth involved in lesions and minimizing surgical defects is highly advantageous to patients.

The rationale for root canal therapy for standing vital teeth roots protruding into the cystic lesion is that surgical intervention causes devitalization of all teeth associated with the cyst (19 , 20). This may lead to persistent periapical infections or immediate or delayed postoperative infection (20). Moreover, peripheral ostectomy to reduce lesion recurrence could cause devitalization of adjacent teeth, as well as lesions involving the teeth. If the lesion is an OKC or unicystic ameloblastoma, a more aggressive peripheral ostectomy is recommended owing to high recurrence rates (16 , 21). Although dentigerous cysts are the most commonly suspected cystic lesions associated with impacted third molars, mandibular OKC and unicystic ameloblastomas are frequently associated with them (22 , 23). Therefore, without a definitive diagnosis of the lesion, surgeons tend to choose a somewhat aggressive peripheral ostectomy, although radiographic findings and clinical features help make a tentative diagnosis. Marsupialization can help maintain the vitality of involved teeth because the part of the cystic lining removed during the procedure aids in making a definitive diagnosis of the lesion. When the pathology reveals less aggressive cysts, such as dentigerous and radicular cysts, the vitality of the involved teeth can be preserved because minimum peripheral ostectomy is recommended. Furthermore, lesion shrinkage over time can allow exclusion of dental roots previously protruded into the cystic cavity from the lesion. We investigated the extent to which involved dental roots could be excluded from the lesion during marsupialization. In the literature, only few studies have evaluated the preservation of tooth vitality after marsupialization of cystic lesions. Diarra et al. reported that, during total marsupialization periods ranging from 6 to 62 months, 84.5% of the teeth involved in lesions were preserved following definitive surgery (24). In the present study, after an average marsupialization duration of 62.51±31.17 weeks, the number of teeth involved in lesions was reduced by 46.48%. The reduction rates at 12, 24 and 36 weeks were 13.44, 26.69, and 30.93%, respectively. Despite performing peripheral ostectomy in this study, 46.48% of the involved teeth ultimately remained unaffected by lesions over the total mean treatment period and had preserved pulp vitality.

Furthermore, we hypothesized that the change in speed of lesion shrinkage over time could help determine the timing of secondary enucleation surgery. The duration of marsupialization has been actively debated. Some authors advocate that sufficient bone formation for enucleation has occurred 6-12 months after marsupialization (18 , 25 - 27); however, some authors advocate for a period of 4 to 6 months (15 , 27 , 28). Among the authors who reported shorter durations, Bodner et al. (29) recommended that cysts be enucleated 3 months after marsupialization. Zhao et al. (14) reported significant increases in bone apposition and a remarkable decrease in the cyst cavity 3 months after decompression. However, in the current study, based on the interval velocity, the most pronounced lesion shrinkage on the radiographs occurred 16 to 24 weeks after marsupialization. This implies that surgery before 16 weeks could be disadvantageous in terms of maximizing new bone formation and preserving tooth vitality through lesion shrinkage.

Moreover, we observed two types of significant lesion shrinkage: Early significant shrinkage and delayed significant shrinkage. In early significant shrinkage, the difference in lesion size between intervals was the most evident in the early visiting intervals and then slowed with extended time. In contrast, in delayed significant shrinkage, a peak difference occurred slightly later than in the early type. Moreover, there was no statistical difference between the two types in terms of age, initial size of the lesion, presence of the cortex of the bony wall, and pathological type; these factors are considered to influence the speed of volume change after marsupialization (6 , 13 , 30). Therefore, delaying the second enucleation surgery after 24 weeks may be a more suitable approach. This is because significant increases in bone volume with a remarkable decrease in the lesion cavity could be obtained by then. Moreover, at least in multiple vital teeth-affected lesions, tooth vitality could be improved because the involved dental roots can be located outside of the lesions during these periods.

This study has several limitations, including a small sample size, irregular radiographic intervals, and a focus on mostly two-dimensional changes in lesion shrinkage patterns.

Nevertheless, our results provide evidence for the optimal duration of marsupialization of cystic odontogenic jaw lesions and the related benefit of preserved teeth vitality. Although marsupialization is an optional treatment modality for large cystic jaw lesions, it helps preserve lesion-involved teeth vitality as well as adjacent anatomical structures, such as the inferior alveolar and infraorbital nerves, nasal cavity, and maxillary sinus, and is thus of potential interest to patients. Furthermore, we assessed the interval speed of shrinkage and evaluated patterns of lesion changes to predict the optimal timing of the secondary definitive surgery. To the best of our knowledge, this is the first attempt to apply such an approach.

## Conclusions

In conclusion, marsupialization is an effective treatment modality for preserving tooth vitality while avoiding damage to adjacent anatomical structures. Lesion shrinkage was the most significant 16-24 weeks after marsupialization. Therefore, the second enucleation surgery should be delayed by 24 weeks to maximize neo-bone formation in the cystic odontogenic jaw lesion cavity and maintain the vitality of lesion-involved and adjacent teeth. Prospective controlled studies with larger sample sizes could help establish the optimal timing for definitive surgery after marsupialization.

## Figures and Tables

**Table 1:** Table Clinical and radiographic data of lesions.

Case	Gender/ Age	Histological type	Total periods of marsupialization (weeks)	Location	Cortical perforation (site)	Relative initial size (AU)	Initial volume (cc)	Initial number of affected tooth	Relative final size and change (%)	Final volume and change (%)	Preserved number of teeth and percentage (%)
Case 1	M/35	RC	89.14	mandible	Yes (L)	592697.68	N.A.	1	255835 (56.84 )	N.A.	1 (100)
Case 2	M/31	RC	42.00	mandible	Yes (B)	373329.39	4.76	3	185700.57 (50.26 )	1.06 (77.65)	1 (33.33)
Case 3	F/47	RC	19.14	maxilla	Yes (B)	228964.28	N.A.	4	168662.24 (26.34)	N.A.	2 (50)
Case 4	M/51	DC	118.57	maxilla	No	643540.60	11.21	8	353132.45 (45.13)	3.77 (66.32)	7 (87.5)
Case 5	M/34	UA	46.00	mandible	No	590325.40	6.39	2	309767.97 (47.53)	1.12 (82.51)	0 (0)
Case 6	M/62	RC	50.00	maxilla	No	643926.83	8.99	4	273638.88 (57.5)	5.47 (39.19)	3 (75)
Case 7	M/48	DC	110.00	mandible	Yes (L)	368797.89	N.A.	1	88237.47 (76.07)	N.A.	1 (100)
Case 8	M/40	OKC	77.14	mandible	Yes (L)	880293.38	N.A.	2	321914.48 (63.43)	N.A.	1 (50)
Case 9	M/33	UA	73.00	mandible	Yes (L)	1035666.40	N.A.	2	300400.23 (70.99)	N.A.	2 (100)
Case 10	M/42	DC	31.00	mandible	No	497113.72	N.A.	0	354646.93 (28.66)	N.A.	0 (0)
Case 11	M/51	RC	31.14	maxilla	Yes (B)	651418.78	8.14	7	303962.31 (53.34)	4.94 (39.28)	4 (57.14)
Case 12	F/52	OKC	104.43	mandible	Yes (B)	2113039.17	N.A.	11	659339.04 (68.8)	N.A.	0 (0)
Case 13	F/39	DC	35.00	maxilla	Yes (B,P)	600360.34	15.22	5	441191.51 (26.51)	4.8 (68.45)	4 (80)
Case 14	F/58	DC	61.00	maxilla	Yes (B)	650284.96	14.53	1	229445.8 (64.72)	2.12 (85.39)	0 (0)
Case 15	F/65	RC	39.00	mandible	No	914973.67	14.96	2	504601.31 (44.85)	6.05 (59.55)	1 (50)
Case 16	M/38	RC	41.29	maxilla	Yes (B)	934768.40	33.95	3	561070.1 (39.98)	6.68 (80.32)	1 (33.33)
Case 17	M/77	DC	118.57	mandible	No	1601587.10	23.78	5	1014308.49 (36.67)	9.06 (61.88)	4 (80)
Case 18	M/50	RC	72.00	maxilla	No	442415.58	N.A.	4	234025.53 (47.1)	N.A.	0 (0)
Case 19	M/47	DC	42.71	maxilla	Yes (B)	1064567.88	22.79	3	338937.72 (68.16)	14.24 (37.49)	1 (33.33)
Case 20	M/48	DC	49.00	mandible	Yes (L)	1022250.45	11.65	1	571809.03 (44.06)	5.09 (56.28)	0 (0)

AU: Arbitrary unit; UA: Unicystic ameloblastoma; DC: Dentigerous cyst; OKC: Odontogenic keratocyst; RC: Radicular cyst; B: Buccal perforation; L: Lingual perforation; P: Palatal perforation; N.A: Not applicable.

## Data Availability

Declared none.
